# Food Insecurity and Micronutrient Status among Ghanaian Women Planning to Become Pregnant

**DOI:** 10.3390/nu12020470

**Published:** 2020-02-13

**Authors:** Ruth Adisetu Pobee, Sixtus Aguree, Esi Komeley Colecraft, Alison D. Gernand, Laura E. Murray-Kolb

**Affiliations:** 1 Department of Nutritional Sciences, The Pennsylvania State University, University Park, PA 16802, USA; 2 Department of Nutrition and Food Science, University of Ghana, 00233 Legon-Accra, Ghana

**Keywords:** micronutrients, food insecurity, women of reproductive age, anemia, iron, retinol, zinc, copper

## Abstract

We examined the association between food insecurity (FIS) and micronutrient status among Ghanaian women planning to become pregnant. A cross-sectional analysis was completed of 95 women aged 18–35 years, living in the Upper Manya Krobo District in the Eastern Region of Ghana. Questionnaires were administered to collect sociodemographic and food security data; weight and height were measured. Blood was drawn from an antecubital vein; one drop was used to assess hemoglobin via Hemocue. Zinc and copper were analyzed using flame atomic spectrophotometry while iron biomarkers, retinol and 25-hydroxyvitamin D were analyzed using ELISA, ultra-performance liquid chromatography and liquid chromatography-tandem mass spectrometry, respectively. Logistic regression models were used to determine the relationship between food insecurity (FIS) and micronutrient deficiencies. FIS was reported among 23% of the households, while micronutrient deficiencies ranged from 7–28% irrespective of FIS status. Retinol concentrations were negatively associated with FIS (*p* = 0.043) after controlling for covariates, although levels were within the normal range in both groups. No statistically significant associations between FIS and micronutrient deficiencies were found. Among those with FIS, 59% were deficient in at least one nutrient with 18% deficient in two nutrients. Unmarried women were at higher risk of FIS (*p* = 0.017) than married women. FIS was associated with retinol concentrations but not other micronutrient biomarkers in Ghanaian women expecting to become pregnant in the next 6 months.

## 1. Introduction

Globally, over two million people are at risk of vitamin and mineral deficiencies, most of whom live in low-income countries [1,2]. Micronutrient deficiencies of particular concern are those of iron, zinc and vitamins A and D, especially among women of reproductive age [3,4]. Women become vulnerable to micronutrient deficiencies during pregnancy as a result of increased demand for growth of the placenta, fetus and maternal tissues [5]. As such, it is challenging for women to consume diets that meet this increased demand. Micronutrient deficiencies have been linked to food insecurity (FIS) [6,7,8,9], a condition associated with inadequate diet and poor nutrient intake as a result of people not having enough food to eat [10]. Thus, FIS has the potential to impact micronutrient status, which, during pregnancy, may lead to poor birth outcomes [11]. The United Nations’ Food and Agriculture Organization estimates that, worldwide, about 850 million people do not have enough to eat [12]. This problem is further exacerbated by factors such as poor dietary quality (especially lack of micronutrient dense foods) and poor micronutrient bioavailability [13].

Several studies have shown lower intakes of energy, protein and some vitamins and minerals among food-insufficient households compared with their food-sufficient counterparts [6,7,8,9]. These studies, however, did not assess blood-based nutrient concentrations, which reflect nutritional status and are less prone to measurement errors when compared to nutrient intakes and dietary recalls [14]. To our knowledge, only two studies to date have specifically examined the relation between FIS and micronutrient status among women of reproductive age. One study, which was conducted in the US, compared dietary intakes and serum nutrient concentrations of adults from families who reported food insufficiency to those who reported food sufficiency [15]. They found that both younger and older adults from FIS families had lower serum concentrations of vitamin A, carotenoids and vitamin E compared with those from food secure (FS) families. The second study was conducted in Tanzania and found no association between FIS and serum ferritin or vitamin A concentrations [16]. The paucity of studies coupled with the discrepant findings highlights the need for more studies that examine the association between FIS and nutritional status biomarkers among women, especially in developing countries. It is important to study women of reproductive age who expect to become pregnant, due to their vulnerability with respect to increased micronutrient demands and the consequences of FIS on the health status of them and their children. This paper examines the association between FIS and serum/plasma micronutrient concentrations for biomarkers of iron, zinc, copper, vitamin A and vitamin D among Ghanaian women expecting to become pregnant in the next 6 months. We hypothesized that women who are FIS will be more likely to be deficient in one or more micronutrients than women who are food secure (FS).

## 2. Materials and Methods

This work utilized data from a pilot study of micronutrient status conducted in Ghana. Ghanaian non-pregnant women between the ages of 18–35 years, who lived in Asesewa (the Eastern Region of Ghana), and were planning to become pregnant within the next 6 months were eligible for this study. Ethical approval was obtained from the ethical review board of the Noguchi Memorial Institute of Medical Research under the University of Ghana and The Pennsylvania State University Institutional Review Board. Women were initially recruited from churches and mosques in the community. Subsequently, a house-to-house recruitment strategy was employed in order to reach the desired sample size. Participants were directed to the Asesewa Government Hospital where data collection occurred. Hospital phlebotomists collected blood samples from an antecubital vein, and trained field personnel administered the questionnaires and took anthropometric measurements. Questionnaires were completed with digital Samsung Galaxy tablets. Blood samples were centrifuged and serum and plasma were aliquoted and initially frozen in a liquid nitrogen tank at the data collection site. Samples were transferred within two weeks to the Noguchi Memorial Institute for Medical Research where they were stored in a −80 °C freezer until they were shipped to The Pennsylvania State University for blood micronutrient analysis. A total of 100 women enrolled in the study. Five women were excluded from this analysis for the following reasons: two women did not know their age or date of birth, two women did not provide FIS and socioeconomic status (SES) data and one woman did not have enough plasma collected for copper analysis. Thus, complete data for 95 women were available for statistical analyses.

### 2.1. Socioeconomic and Demographic Variables

Sociodemographic variables such as age (dichotomized as 18–23 and 24–35 years based on work by Garcia [17]), marital status defined in the Ghanaian context as any couple either married or coexisting or cohabitating (dichotomized as married or not), level of education (categorized as no school, 1–9 years of schooling and >9 years of schooling), parity (dichotomized as 0 or ≥1 child), electricity availability in the household (dichotomized as yes or no) and SES (divided into three groups, lowest 40%, middle 40%, highest 20%) were collected and categorized as indicated for analysis.

### 2.2. Household Food Insecurity

The eight items comprising the US Adult Food Security Scale [17] on the 18-item US Household Food Security Survey Module [18] were used to measure household food insecurity over the past month (see Table S1: for Food Insecurity Questionnaire). Factor analysis was applied to understand the psychometric behavior of the original eight adult items. All eight items had adequate psychometric behavior and scores loaded adequately on one factor with internal consistency on all items being above 0.9. Thus, household food insecurity was dichotomized as either food secure (score = 0 on all eight items) or not (score ≥ 1).

### 2.3. Anthropometry

Weight was taken to the nearest 0.1 kg using the Seca 874 flat scale (Seca GmbH, Hamburg Germany), which was calibrated weekly with known weights, while height was measured to the nearest 0.1 cm using the Seca 217 stadiometer (Seca GmbH). Individuals were in light clothing and all extra clothes, bangles, earrings, phones, etc. were removed before weight measurements were taken. Both weight and height measurements were done in duplicate and the mean was used. BMI was calculated as weight (kg)/height (m^2^) and was classified as normal (≤24.9 kg/m^2^), overweight (25.0–29.9 kg/m^2^) or obese (≥30 kg/m^2^) using WHO cutoffs [19].

### 2.4. Blood Collection and Laboratory Analyses

In short, about 8 mL of blood were drawn into plasma and serum collection tubes (4 mL each) by a trained phlebotomist using standard procedures [20]. Hemoglobin concentration was determined immediately via a Hemocue (HB201; HemoCue America, Brea, CA, USA). The remaining blood was centrifuged using standard procedures and plasma and serum samples were aliquoted into five microcentrifuge tubes each. Plasma samples for retinol analysis were covered with foil to prevent vitamin A degradation from light. Serum ferritin (Ft) was analyzed using an immunoassay procedure (Ramco Laboratories Inc., Strafford, TX, USA), while serum iron (Fe) and total iron binding capacity (TIBC) were analyzed using colorimetric methods [21]. Plasma zinc (Zn) and copper (Cu) were determined using flame atomic absorption spectrophotometry [22]. Plasma retinol concentrations were measured using ultra-performance liquid chromatography (UPLC). Measurements of serum 25(OH)D_3_ and 3-epi-25-hydroxyvitamin D_3_ (3-epi-25(OH)D_3_) were performed at the Analytical Facility for Bioactive Molecules, The Hospital for Sick Children (Toronto, ON, Canada) using liquid chromatography-tandem mass spectrometry [23]. The inflammatory markers alpha-1-acid-glycoprotein (AGP) and C-reactive protein (CRP) were measured using radial immunodiffusion tests (Kent Laboratories Inc., Bellingham, WA, USA). The following cut off values were used to categorize women as deficient: Hb < 12 g/dL, Ft < 15 µg/L [24], transferrin saturation (TSAT) < 16% (calculated as (Fe/TIBC ×100)) [25], and retinol < 20 μg/dL [26]. Cu < 90 µg/dL [27], Zn < 70 µg/dL [28], and total 25(OH)D_3_ {25(OH)D_3_ + 3-epi-25(OH)D_3_} < 50 nmol/dL [29] were used to indicate insufficiency. Although some cutoff points represent insufficiency, for simplicity, deficiency or insufficiency are collectively called deficiency here. Women were categorized as inflamed if CRP was >5 mg/L and/or AGP was >100 mg/dL [30] and correction factors for Ft, Zn and retinol were applied as in our previous work [31]. For more details on blood collection and processing see Gernand et al. [31].

### 2.5. Sample Size

The purpose of the main study was to determine whether or not it was feasible to recruit and follow 100 women who expected to become pregnant in the ensuing 6 months [31]. As such, the study was not originally powered to examine micronutrient deficiencies based on food security status. Our power to detect significant differences in nutrient levels between FS and FIS groups ranged between 5.9% (zinc) to 58.5% (retinol).

### 2.6. Data Analyses

SAS version 9.4 was used for data analyses. The prevalence rates of FIS, single micronutrient deficiencies, and multiple micronutrient deficiencies were calculated based on frequencies. In order to get the prevalence of simultaneous multiple micronutrient deficiencies, we first categorized each nutrient biomarker (zinc, copper, retinol, 25(OH)D_3_ and ferritin) as deficient (score = 1) or not deficient (score = 0) based on the cut offs described earlier. We then added all nutrient categories for each individual to obtain a total score. This total score was then used in subsequent analyses to represent the number of simultaneously occurring nutrient deficiencies. We also examined continuous variables for normality of the distribution. All variables were normally distributed, except ferritin, which was log transformed.

We calculated an SES index using principal component analyses (PCA). SES variables were dichotomized as 1 = yes and 0 = no. Those with <10% or >90% endorsement were excluded (household electricity usage, ownership of animal drawn cart, materials used for wall of dwelling, roofing material, flooring material and source of water), due to their low contribution to the SES index [32]. Variables with low frequencies (e.g., use of covered or open well, use of Kumasi ventilated improved pit or public toilet) were combined. Variables with large loadings (weight ≥ 0.1 or ≤−0.1) were retained in the final 16 asset model. Factor scores were calculated for each household based on the eigenvectors (weights) from the first principal component by multiplying the eigenvectors (coefficients) by the item value (1 or 0) and summing them to obtain the factor score for each household. The factor score was then used as the household socio-economic score. The higher the household socio-economic score, the higher the SES for that household. We then used the 40:40:20 approach to classify households into lowest, middle and highest SES, respectively [33]. The following variables were retained in the model: radio, TV, fridge, video deck, DVD player, sewing machine, bicycle, car/truck, agricultural land, livestock, occupancy status of household and renting of land or building for business (each asset was categorized as owned by the household or not). Our goal was to capture as much information as possible that reflected long term household SES [34].

A Fisher exact test was used to examine bivariate differences in maternal characteristics (marital status, age, parity, BMI, SES index, years of schooling) and micronutrient concentrations between women who were food insecure and those who were food secure.

Generalized linear models (GLM) were used to determine the differences between the mean micronutrient concentrations of FS and FIS households. Logistic regression models were employed to determine the overall relationship between FIS and single as well as multiple micronutrient deficiencies. Odds ratios and their respective 95% CI were calculated. Potential confounders identified from the literature and that showed a significant correlation with FIS in our data were controlled for. Three sets of regression models were run as follows: (1) a bivariate model including only FIS status as the exposure and individual micronutrient concentrations as the outcome; (2) model 1 plus adjustments for marital status, age, parity, BMI, SES and years of schooling; and (3) model 2 plus adjustments for all the other micronutrient concentrations (zinc, copper, ferritin, TSAT, retinol and two forms of vitamin D {25(OH)D_3_ or 25(OH)D_3_ + 3-epi-25(OH)D_3_}) except the nutrient being used as the outcome variable in the model. We reported the mean and standard error and set the significance level at *p* < 0.05.

## 3. Results

Sociodemographic and micronutrient status variables are shown in Table 1. Overall, there was a 23% prevalence of FIS among the women studied. The mean BMI was 25.0 ± 6.0 kg/m^2^ for FIS women and 25.9 ± 5.2 kg/m^2^ for FS women (*p* = 0.546). Nearly 33% had anemia and the prevalence of deficiency for each micronutrient ranged from 7–28%. In terms of inflammation, 4% of women had a high CRP and 11% of women had a high AGP. There were no meaningful associations between FIS and any of the socio-demographic variables considered, except marital status. Compared to married women, more unmarried women were food insecure (*p* = 0.017). For four of the biomarkers (hemoglobin, ferritin, TSAT and 25(OH)D_3_), the prevalence of deficiency was larger in those women who were FIS versus those who were FS (although the differences were not significant). Of those who were FIS, 18% were deficient in at least two nutrients compared to 14% of those who were FS (non-significant difference). When we analyzed for co-occurring nutrient deficiencies based on FIS status, we found that 59% of FIS women were deficient/insufficient in at least one or more nutrients, while 18% were deficient/insufficiency in at least two or more nutrients. No woman was deficient in three or more nutrients.

Table 2 shows the mean micronutrient concentration of the women by food insecurity status and indicates that the mean for each nutrient was well within the normal range. Among all the micronutrients studied, only retinol concentrations showed statistically significantly higher levels in FS compared to FIS women both before and after controlling for confounders. However, the retinol concentration of both groups were largely within the normal range. Zn, Cu, Ft, TSAT and 25(OH)D_3_/3-epi-25(OH)D_3_ concentrations did not differ between FS and FIS groups either before or after adjusting for potential confounders, including marital status, age, parity, BMI, SES and years of schooling.

The odds of micronutrient deficiencies by FIS status (Table 3) did not differ for single or multiple micronutrient deficiencies (adjusted and unadjusted models). Interestingly, the odds of copper deficiency for FIS vs FS was much higher in the fully adjusted model 3 compared to models 1 and 2, although the results were not statistically significant. For several nutrients, the association moved away from the null after adjustments, but remained non-statistically significant.

## 4. Discussion

Micronutrient deficiencies remain a global challenge to the health of reproductive age women and may be consequences of food insecurity and a poor diet. In this study, we explored the association between food insecurity and blood-based concentrations of selected micronutrients among Ghanaian women expecting to become pregnant in the ensuing 6 months. There was a negative relation between FIS and plasma retinol concentrations but no relation found with any of the other micronutrients tested, nor with micronutrient deficiencies.

Previous work indicates that, for households to be food secure, the following conditions must be met: physical availability of food, economic and physical access to food and adequate food utilization that relies heavily on the ability of the body to process/use nutrients as well as on dietary quality and the safety of the foods consumed [13]. The food security problem in Africa has been described as worrisome because the population in Africa is expected to increase to 2 billion by 2050 and it is uncertain how to provide food for that many people [37]. Poverty and food shortage have been found to be the main catalysts to food insecurity. The prevalence of FIS has been reported to be 60% among South African women in their postpartum period [38] and 32% among women in Northern Jordon [39]. Another study also found the prevalence of FIS to be 72% among Ghanaian adolescents girls and 83% among young adults in South Africa [40], while a study among rural women in Kenya reported FIS rates of 48% [41]. These prevalence rates are higher than what we observed in our population where the problem of food insecurity was found to be of moderate public health concern (23%).

Our hypothesis that food insecurity would be associated with micronutrient status was not supported by these findings. Food insecurity was not significantly associated with serum concentrations of iron, copper, zinc or 25(OH)D_3_, nor was it associated with single or multiple deficiencies. However, food insecurity was associated with lower serum retinol compared to food security. Our findings agree with those of Dixon et al. [15], who found a difference in serum vitamin A levels between FS and FIS individuals. Our findings are also in partial agreement with the findings of Leyna et al. [16] in that both our plus their findings indicated no relation between FIS and serum ferritin; however, we differ in our findings of the relations between FIS and plasma retinol. In our study, we adjusted for multiple variables that were related to FIS, including micronutrient concentrations, but there was no indication that these adjustments were done in the study conducted by Leyna et al. [16]. Additionally, our sample size and the prevalence of vitamin A deficiency was low in our population, compared to that of Leyna et al. This may account for the difference in results. One implication of our findings of no associations with most of the nutrients studied could be that FIS may not be a good proxy or screening tool for assessing the risk of multiple micronutrient deficiencies. The FIS questionnaire used here is an eight-item questionnaire [17] adapted from the US-household food security survey [18]. The questions address concerns about food quantity and quality during the last month. Examples of the questions include; “How often was the following statement true? I worried whether our food would run out because of lack of money or other resources to get food”, “Did you or the other adults in your household ever eat less, consume less preferred foods, feel hungry but did not eat or skip meals because there wasn’t enough money for food?”, “Were you ever hungry but did not eat because of lack of money or other resources to buy food?” [42]. The questions are qualitative in nature and depend on the individual’s perception. Some of these questions (reduction of food intake and consuming less preferred foods) may reflect coping mechanisms [43] that these individuals have adopted to deal with their situation of FIS and they may have gotten used to them. Therefore, the individuals may not be perceiving FIS and not report it as such. Thus, answers to these questions may not truly capture FIS and are unlikely to reflect the body’s levels of nutrients.

Out of all the sociodemographic variables analyzed, only marital status was significantly associated with FIS, with significantly more unmarried women experiencing FIS than married women. Several other studies have indicated a lower prevalence of FIS in those who are married [16,44] with possible reasons being that marriage may enhance family income and wealth and provide social support and other noneconomic resources [45,46,47], thus making married women more food secure. In other studies, however, this relation was not observed [41,48].

Our study was limited by several factors. First, our sample was from one town and was a group of women who intended to become pregnant in the ensuing 6 months and, as such, may not be applicable to other women in Ghana. Second, our study used a small sample size and was not originally powered to look at the relation between FIS and micronutrient deficiencies. Third, this study was cross-sectional and, as such, is unable to establish a temporal relation. This study also lacked a measure of habitual intake which limits our ability to interpret the reason for the micronutrient deficiencies that were measured. Another limitation is that FIS was measured with an eight-item questionnaire adapted from the US household food security survey. This questionnaire may not be the best suited to estimate FIS in this population. A more comprehensive assessment and long-term measure of FIS that could be beneficial would consider food purchases, food patterns, food habits, consumption patterns, purchasing power, food quantity and quality as well as coping mechanisms [43]. Seasonal changes in food availability could affect household food insecurity. In order to capture the actual FIS situation in this population, there is the need to assess FIS during periods of food scarcity (“lean season”—March to July in southern Ghana) and food abundance. Our study, however, was conducted primarily during periods of food availability and a very small period of the lean season.

The main strength of this study was the use of five different serum/plasma micronutrient biomarker concentrations in relation to FIS status. To our knowledge, this is the first study to look at several micronutrients in relation to FIS among women expecting to become pregnant. This study therefore adds to the literature on micronutrient deficiencies and FIS. Another strength of this study is our sample population, women who intend to become pregnant in the next 6 months, a population that is rarely studied yet whose nutritional status is an important risk factor for adverse pregnancy outcomes.

## 5. Conclusions

Our findings show that among Ghanaian women expecting to become pregnant, there was a significant association between plasma retinol concentrations (which were within the normal range in both groups) and FIS, but no other micronutrient status/concentrations, even after adjusting for covariates. We also found a significant association between marital status and FIS with unmarried women being at a higher risk of FIS. The finding of no relation between micronutrient status using traditional cutoff points and food insecurity was surprising but may be attributable to our small sample size or the inability of the FIS questionnaire to adequately capture food insecurity in this population. The prevalence of micronutrient deficiencies ranged from 7–28% irrespective of FIS status. Given the importance of micronutrients during pregnancy and the potential association between FIS and micronutrient status, it will be important to conduct studies with larger sample sizes in order to truly understand this relation in women who are expecting to become pregnant.

## Figures and Tables

**Table 1:** Sociodemographic variables and micronutrient status of Ghanaian women categorized by food security status.

	Food Secure *n* = 73		Food Insecure *n* = 22		X^2^	*p*-Value
	*n*	%	*n*	%		
**Age (years)**						
18–24	28	38.4	10	45.4	0.35	0.551
25–35	45	61.6	12	54.6		
**BMI**						
Normal (≤24.9 kg/m^2^)	41	56.2	16	72.7	2.73	0.256
Overweight (≥25 ≤ 29.9 kg/m^2^)	18	24.7	2	9.1		
Obese (≥30 kg/m^2^)	14	19.2	4	18.2		
**Years of Schooling**						
0–6 years	12	16.4	3	13.6	0.28	0.786
7–9 years	29	39.7	8	36.4		
>9 years	32	43.8	11	50.0		
**SES Index ^a^**						
Lowest (40%)	26	35.6	14	63.6	5.55	0.062
Middle (40%)	32	43.8	6	27.3		
Highest (20%)	15	20.6	2	9.1		
**Married**	34	46.6	4	18.2	5.68	0.017 *
**Parity**						
No child	32	43.8	12	54.5	0.78	0.377
1–4 children	41	56.2	10	45.5		
**Nutritional Biomarkers**						
Hemoglobin < 12 g/dL	22	30.1	9	40.9	0.89	0.345
Adjusted Ferritin ^b^ < 15 μg/L	7	9.6	4	18.2	1.22	0.270
TSAT < 16%	19	26.0	8	36.4	0.89	0.346
Adjusted Retinol ^c^ < 20 µg/dL	6	8.2	1	4.6	0.33	0.563
Adjusted Zinc ^d^ < 70 µg/dL	12	16.4	3	13.6	0.10	0.752
Copper < 90 µg/dL	11	15.1	1	4.6	1.69	0.193
25(OH)D_3_ < 50 nmol/L ^e^	8	11.0	5	22.7	1.98	0.159
25(OH)D_3_ + 3-epi-25(OH)D_3_ <50 nmol/ ^e^	5	6.9	3	13.6	1.01	0.315
**Co-occurring Nutrient Deficiencies with TSAT as Iron biomarker ^f^**						
Deficient in at least one nutrient	39	54.4	13	59.1	0.22	0.640
Deficient in at least two nutrients	10	13.7	4	18.2	0.27	0.603
**Co-occurring Nutrient Deficiencies with Ferritin as Iron biomarker ^f^**						
Deficient in at least one nutrient	31	42.5	11	50.0	0.39	0.533
Deficient in at least two nutrients	9	12.3	3	13.6	0.03	0.871

BMI: Body mass index. SES: socioeconomic status. TSAT: Transferrin saturation. ^a^ SES Index was calculated based on ownership of the following items: radio, TV, fridge, video deck, DVD player, sewing machine, bicycle, car/truck, agricultural land, livestock’s, occupancy status of household, renting of land or building for business. Principal component analysis was performed and divided into three groups; lowest 40%, Middle 40%, Highest 20%. ^b^ Adjustment using correction factors from Thurman et al. [30]. ^c^ Adjustment using correction factors from Thurnham et al. [35]. ^d^ Adjustment using correction factors from Mburu et al. [36]. ^e^ No participant had a value < 30nmol/L for 25(OH)D_3_; the range defined as insufficiency was between 30–50 nmol/L, ^f^ Co-occurring nutrient deficiencies out of the five nutrients studied (iron, zinc, copper, retinol and 25(OH)D_3_), * Significant *p* < 0.05.

**Table 2:** Mean and standard error (SE) of micronutrient concentrations among Ghanaian women expecting to become pregnant in food secure and food insecure households.

Micronutrient Concentrations	Model 1		Model 2		Model 3	
	Mean	SE	Mean	SE	Mean	SE
**Zinc ^a^ (g/dL)**						
FS	82.8	1.6	82.6	1.7	82.5	1.6
FIS	81.1	2.9	81.9	3.1	82.1	3.1
*p*-value	0.626		0.844		0.905	
**Copper (µg/dL)**						
FS	114.4	2.9	114.1	3.0	113.9	3.0
FIS	112.9	5.3	114.2	5.7	114.7	5.8
*p*-value	0.787		0.983		0.907	
**Retinol ^b^ (µg/dL)**						
FS	39.4	1.7	39.5	1.7	39.5	1.6
FIS	32.4	3.0	32.0	3.2	32.1	3.1
*p*-value	0.043 *		0.046 *		0.046 *	
**25(OH)D_3_** **(nmol/L)**						
FS	65.6	1.7	65.7	1.7	65.5	1.7
FIS	63.3	3.1	63.3	3.3	64.1	3.4
*p*-value	0.566		0.532		0.737	
**25(OH)D_3_ +** **3-epi-25(OH)D_3_ (nmol/L)**						
FS	68.4	1.8	68.5	1.8	68.3	1.9
FIS	66.8	3.3	66.5	3.5	67.3	3.6
*p*-value	0.673		0.628		0.830	
**Iron (TSAT) (%)**						
FS	26.0	1.6	25.4	1.6	24.9	1.6
FIS	24.0	2.9	26.1	3.1	27.5	3.1
*p*-value	0.559		0.826		0.484	
**Iron (Ft) ^c^ (µg/L)**						
FS	74.5	6.8	71.7	6.8	71.0	6.8
FIS	64.4	12.5	73.6	13.0	76.0	13.2
*p*-value	0.482		0.902		0.747	

FS: Food security. FIS: Food insecurity. TSAT: Transferrin saturation. Ft: Ferritin. Linear regression models with concentrations as outcome variables. Model 1 no adjustment for any covariates, Model 2 adjusted for marital status, age, parity, BMI, SES and years of schooling. Model 3 adjusted for marital status, age, parity, BMI, SES, years of schooling and all other nutrients (zinc, copper, iron (TSAT or Ft), retinol and vitamin D: {25(OH)D_3_ or 25(OH)D_3_ + 3-epi-25(OH)D3}) except the nutrient used as dependent variable. ^a^ Adjustment using correction factors from Mburu et al. [37]. ^b^ Adjustment using correction factors from Thurnham et al. [35]. ^c^ Adjustment using correction factors from Thurnham et al. [30]. * Significant *p*-value < 0.05.

**Table 3:** Odds Ratios (OR) and 95% Confidence Intervals (CI) of micronutrient deficiencies or insufficiencies according to food security status among Ghanaian women expecting to become pregnant.

Nutrient Deficiency	Model 1 OR (95% CI)	Model 2 OR (95% CI)	Model 3 OR (95% CI)
**Zinc Deficiency**			
FS (reference)	1	1	1
FIS	1.25 (0.32, 4.88)	2.07 (0.46, 9.23)	2.29 (0.49,10.63)
*p*-value	0.752	0.341	0.292
**Copper Deficiency**			
FS (reference)	1	1	1
FIS	3.73 (0.45, 30.61)	6.67 (0.67, 66.0)	13.7 (0.93, 203.11)
*p*-value	0.221	0.105	0.067
**Retinol Deficiency**			
FS (reference)	1	1	1
FIS	1.88 (0.21, 16.52)	2.04 (0.18, 23.27)	2.50 (0.15, 40.58)
*p*-value	0.569	0.569	0.520
**25(OH)D_3_** **Deficiency**			
FS (reference)	1	1	1
FIS	0.42 (0.12, 1.44)	0.48 (0.11, 2.14)	0.45 (0.09, 2.31)
*p*-value	0.168	0.334	0.339
**25(OH)D_3_ +** **3-epi-25(OH)D_3_ Deficiency**			
FS (reference)	1	1	1
FIS	0.47 (0.10, 2.13)	0.49 (0.08, 2.96)	0.71 (0.11, 4.71)
*p*-value	0.324	0.436	0.725
**Iron Deficiency (TSAT)**			
FS (reference)	1	1	1
FIS	0.62 (0.22, 1.70)	0.95 (0.28, 3.05)	1.31 (0.37, 4.70)
*p*-value	0.348	0.931	0.680
**Iron Deficiency (Ft)**			
FS (reference)	1	1	1
FIS	0.48 (0.13, 1.81)	0.69 (0.16, 3.03)	0.76 (0.16, 3.57)
*p*-value	0.277	0.626	0.732
**One or More Nutrient Deficiencies/Insufficiencies**			
FS (reference)	1	1	
FIS	0.79 (0.30, 2.09)	1.38 (0.42, 4.54)	
*p*-value	0.640	0.594	-
**Two or More Nutrient Deficiencies/Insufficiencies**			
FS (reference)	1	1	
FIS	0.71 (0.20, 2.55)	1.05 (0.26, 4.33)	
*p*-value	0.604	0.945	-

FS: Food security. FIS: Food insecurity. TSAT: Transferrin saturation. Ft: Ferritin. Logistic regression models; Model 1 includes FIS, Model 2 adjusted for marital status, age, parity, BMI, SES and years of schooling. Model 3 adjusted for marital status, age, parity, BMI, SES, years of schooling and all nutrients (zinc, copper, iron (TSAT or Ft)), retinol and two forms of Vitamin D: {25(OH)D_3_ or 25(OH)D_3_ + 3-epi-25(OH)D_3_}) except the nutrient used as dependent variable.

## Supplementary Materials

The following are available online at https://www.mdpi.com/2072-6643/12/2/470/s1, Table S1: Food Security Questionnaire.
