# Supplementary material for: Food Insecurity and Micronutrient Status among Ghanaian Women Planning to Become Pregnant
Source: Nutrients. 2020 Feb 13;12(2):470. doi: 10.3390/nu12020470 (PMC7071299; doi:10.3390/nu12020470)
Supplement: Supplementary file 1 [file nutrients-12-00470-s001.pdf]

Supplementary Materials:

Table S1: Food Security Questionnaire.

		Yes	No	Don't know	Refused
1.	During the last month, were you worried that your household would run out of food because of lack of money or other resource to get food?	❍	❍	❍	❍
2.	During the last month, did your household run out of food because of lack of money or other resource to get food?	❍	❍	❍	❍
3.	During the last month, did your household lack enough money or other resource to get healthy and nutritious food?	❍	❍	❍	❍

The next 5 questions ask about adults in the household, referring to persons over 18 years of age.

		Yes	No	Don't know	Refused
4.	During the last month, did you or any adult in your household have to consume a diet based on only few kinds of foods because of lack of money or other resources to get food?	❍	❍	❍	❍
5.	During the last month, did you or any adult in your household not eat breakfast, lunch or dinner [or skip a meal] because of lack of money or other resources to get food?	❍	❍	❍	❍
6.	During the last month, did you or any adult in your household eat less than you thought you should because of lack of money or other resources to get food?	❍	❍	❍	❍
7.	During the last month, did you or any adult in your household feel hungry but did not eat because of lack of money or other resources to get food?	❍	❍	❍	❍
8.	During the last month, did you or any adult in your household eat only one meal in a day or go without eating for a whole day because of lack of money or other resources to get food?	❍	❍	❍	❍
